# Correction: Kumar et al. A Novel Decentralized Blockchain Architecture for the Preservation of Privacy and Data Security Against Cyberattacks in Healthcare. *Sensors* 2022, *22*, 5921

**DOI:** 10.3390/s25154597

**Published:** 2025-07-25

**Authors:** Ajitesh Kumar, Akhilesh Kumar Singh, Ijaz Ahmad, Pradeep Kumar Singh, Anushree, Pawan Kumar Verma, Khalid A. Alissa, Mohit Bajaj, Ateeq Ur Rehman, Elsayed Tag-Eldin

**Affiliations:** 1 Department of Computer Engineering and Applications, GLA University, Mathura 281406, Indiaanushree.gla@gla.ac.in (A.); 2 Institute of Computer Sciences and Information Technology (ICS/IT), The University of Agriculture, Peshawar 25130, Pakistan; 3 Department of Computer Science Engineering, MIT Art, Design and Technology University, Pune 412201, India; 4 SAUDI ARAMCO Cybersecurity Chair, Networks and Communications Department, College of Computer Science and Information Technology, Imam Abdulrahman Bin Faisal University, P.O. Box 1982, Dammam 31441, Saudi Arabia; 5 Department of Electrical and Electronics Engineering, National Institute of Technology Delhi, Delhi 110040, India; 6 Department of Electrical Engineering, Graphic Era (Deemed to be University), Dehradun 248002, India; 7 College of Internet of Things Engineering, Hohai University, Changzhou 213022, China; 8 Faculty of Engineering and Technology, Future University in Egypt, New Cairo 11835, Egypt

## Affiliation Correction

In the published publication [1], there was an error regarding the affiliation “Shenzhen College of Advanced Technology, University of Chinese Academy of Sciences (UCAS), Shenzhen 518055, China”, for Ijaz Ahmad due to improper authorization. The updated affiliation is as follows: “Institute of Computer Sciences and Information Technology (ICS/IT), The University of Agriculture, Peshawar 25130, Pakistan”.

## Text Correction

Reference [10] and Reference [14] were retracted. A correction has been made to Section 1, Paragraph 9. We have updated the sentence “Some applications that integrate and support blockchain technology in the field of EMR and EHR are MedRec [3], FHIRChain [9], Medshare [10], Ethereum applications, MedBlock [11], BlockHIE [12], Ancile [13], and OmniPHR [14].” The sentence has been updated to “Some applications that integrate and support blockchain technology in the field of EMR and EHR are MedRec [3], FHIRChain [9], Ethereum applications, MedBlock [10], BlockHIE [11], and Ancile [12].”

In Section 1, Paragraph 10, we have updated the sentence “MiStore [18] is considered a medical and health insurance system using blockchain that secures and encrypts health-related data [1,9,10,14,18,19,20,21,22].” The sentence has been updated to “MiStore [16] is considered a medical and health insurance system using blockchain that secures and encrypts health-related data [1,9,16,17,18,19,20].”

## References Correction

References [10,14] have been removed. With this correction, the order of some references has been adjusted accordingly.

The authors state that the scientific conclusions are unaffected. This correction was approved by the Academic Editor. The original publication has also been updated.
